# Saudi Moumouvirus, the First Group B Mimivirus Isolated from Asia

**DOI:** 10.3389/fmicb.2016.02029

**Published:** 2016-12-20

**Authors:** Leena H. Bajrai, Felipe L. de Assis, Esam I. Azhar, Priscilla Jardot, Catherine Robert, Jônatas Abrahão, Didier Raoult, Bernard La Scola

**Affiliations:** ^1^Department of Biochemistry, Faculty of Science, King Abdulaziz UniversityJeddah, Saudi Arabia; ^2^Unité des Rickettsies, URMITE UMR CNRS 7278 IRD 198 INSERM U1095, Facultés de Médecine et de Pharmacie, IHU Méditerranée Infection, Aix-Marseille UniversitéMarseille, France; ^3^Laboratório de Vírus, Belo Horizonte, Departamento de Microbiologia, Instituto de Ciências Biológicas, Universidade Federal de Minas GeraisMinas Gerais, Brazil; ^4^Special Infectious Agents Unit, King Fahd Medical Research Center, and Department of Medical Laboratory Technology, Faculty of Applied Medical Sciences, King Abdulaziz University, JeddahSaudi Arabia

**Keywords:** mimivirus, giant viruses, moumouvirus, Saudi Arabia, genome

## Abstract

The number of novel giant viruses identified and characterized from the recently proposed order *Megavirales* has increased in recent years and new questions have been raised regarding viral diversity and evolution. Here, we describe the isolation and characterization of Saudi moumouvirus (SDMV), a new giant virus belonging to *Mimivirus* lineage B, isolated from a sewage sample taken from the King Abdulaziz University hospital in Jeddah, Saudi Arabia. SDMV presented 500 nm icosahedral particles with a 1,046,087 bp genome, which is larger than moumouvirus-like genomes which have been described in the past. The SDMV genome was predicted to encode 868 ORFs, ranging in size from 54 to 2,914 amino acids, with a mean size of 349 aa. Furthermore, this genome was predicted to encode 40 new genes (ORFans) without similarity with other sequences (ORFan L850 transcript was detected by qPCR in infected amoeba), in addition to 42 hypothetical proteins (pseudo-ORFs) with less than 100 aa, which matched other sequences in the NCBI nr database. Phylogenetic analysis showed that SDMV clustered together with mimiviruses from lineage B, including moumouvirus-like strains. It is, therefore, the third Mimivirus to be isolated in Asia and the first of group B.

## Introduction

Free-living amoebae are unicellular phagocytes, which are widely distributed throughout the environment and are found in the soil, water and animals, including humans (Rodriguez-Zaragoza, 1994). The amoebae displays macrophage behavior, although a particular micro-organism marker recognition is not necessary due its ability to phagocyte all particles larger than 0.5 μm (Audic et al., 2007). However, some of those micro-organisms resist digestion by amoebae after phagocytosis (Greub and Raoult, 2004). The discovery in La Scola et al. (2003) of the first giant virus, named *Acanthamoeba polyphaga mimivirus* (APMV), enabled some aspects of digestion-resistant environmental viruses to be understood. The APMV is the founder member of family *Mimiviridae* and possesses a linear 1.2 Mbp dsDNA genome, located in the innermost part of a large (500 nm) icosahedral capsid which is coated by a surrounding layer of fibrils (Raoult et al., 2004; Xiao et al., 2005; Arslan et al., 2011; Colson et al., 2012). Some aspects of its biology, such as complex and large virions, replication cycle features, in addition to the genome content and phylogeny, place the members of the family *Mimiviridae* into the nucleocytoplasmic large DNA virus (NCLDV) group (La Scola et al., 2003; Raoult et al., 2004; Iyer et al., 2006; Yutin et al., 2009; Mutsafi et al., 2010). Colson et al. (2012) have proposed reclassifying giant viruses into a new order *Megavirales*, which consist of members of the viral families *Iridoviridae*, *Phycodnaviridae*, *Asfarviridae*, *Ascoviridae*, *Poxviridae* and the recently discovered families *Mimiviridae* and *Marseilleviridae* (Iyer et al., 2006; Colson et al., 2012, 2013).

Recently, members of family *Mimiviridae* have been associated with pneumonia in humans, increasing concerns about the diversity and widespread nature of these organisms. (La Scola et al., 2003, 2008; Raoult et al., 2007; Saadi et al., 2013). Over the last decade, the isolation and characterization of new members of the family *Mimiviridae* from different regions and sources have been reported, allowing some aspects about the phylogeny of this family to be elucidated (Colson et al., 2012; Campos et al., 2014; Mozar and Claverie, 2014; Assis et al., 2015).

Recently, it was proposed to cluster mimiviruses into two genera: (1) *Mimivirus*: sub-divided into three non-taxonomical groups based on polB sequences: Group A (APMV and *Mamavirus*), Group B (Moumouvirus), Group C (*Megavirus chilensis*); and (2) *Cafeteriavirus*, which is a distant relation of the family *Mimiviridae* (Fischer et al., 2010; Arslan et al., 2011; Colson et al., 2012).

In this paper, we describe the isolation of a new member of the genus *Mimivirus*, Group B (Moumouvirus-like), from sewage samples collected from the King Abdulaziz University Hospital in Jeddah, Saudi Arabia. By using an amoeba-associated virus co-culture (*A. polyphaga)* as previously described (La Scola et al., 2001), this new virus detected and named the SDMV. We present some biological characteristics of SDMV and the main features observed through sequencing its complete genome.

## Materials and Methods

### Isolation and Presumptive Identification of Virus

A total of eight samples were collected from the public sewers of Jeddah: four before and four after treatment. Nine samples were camel stools. Samples were prepared as described previously (La Scola et al., 2001). The following amoebae were inoculated and incubated for 2 days under the conditions previously described (La Scola et al., 2001): *A. polyphaga* (strain Linc AP-1), *Acanthamoeba griffini* (strain ATCC50702), *Acanthamoeba* sp. (strain E4), *Vermamoeba vermiformis* (strain CDC19), and *Dictyostelium discoideum* (Strain ATCC44841). The following mixture of antibiotics was used to avoid bacterial overgrowth: Vancomycin (10 μg/ml), Imipenem (10 μg/ml), Doxycycline (10 μg/ml), Ciprofloxacin (20 μg/ml), and Thiabendazole (30 μg/ml). Sub-cultures were then performed every 48 h using the same conditions described above on fresh amoebae without any antibiotics. These were screened daily by inverted microscope under 400X magnification for cytopathogenic effects (La Scola et al., 2001). The size and morphology of the viral particles in wells showing amoeba lysis were determined by electron microscopy using negative staining (La Scola et al., 2010). SDMV was produced within the viral factory located in the *A. polyphaga* cytoplasm and stained with 4,6-diamidino-2-phenylindole (DAPI) and hemacolor. In order to work on a clonal isolate, culture supernatant containing viral particles was serially diluted from 10^-1^ to 10^-11^. Three 24-well microplates were prepared by adding 500 μl of suspension containing 5 × 10^4^ rinsed amoebae and four wells were inoculated with each viral dilution. Until the lysis of amoebae was almost complete at 32°C, the microplates were observed daily under an inverted microscope. The well-containing the highest dilution, which caused complete lysis, was used for virus production through consecutive sub-culturing (La Scola et al., 2010). The automated EZ1 Virus MiniKit was used to extract viral DNA. Five real-time PCR systems based on hydrolysis probes for detecting the viral groups (mimivirus lineages A, B, and C; marseilleviruses; virophages) were applied and conducted as previously described (Ngounga et al., 2013).

### Genome Sequencing and Assembly

The SDMV genome was sequenced using the Illumina MiSeq instrument (Illumina, Inc., San Diego, CA, USA) with the paired-end strategy in line with the manufacturer’s recommendations for the Nextera XT library kit (Illumina). To do so, the extracted genome of SDMV was quantified using a Qubit assay with a high sensitivity kit (Life Technologies, Carlsbad, CA, USA) to 48.8 ng/μl, and the dilution was performed, requiring 1 ng of DNA as input. The ‘tagmentation’ step fragmented the genomic DNA. Limited cycle PCR amplification then completed the tag adapters and introduced dual-index barcodes. After purification on AMPure beads (Life Technologies, Carlsbad, CA, USA), the libraries were then normalized on specific beads according to the Nextera XT protocol (Illumina). Normalized libraries were pooled into a single library for sequencing on the MiSeq. The pooled single strand library was loaded onto the reagent cartridge and then onto the instrument along with the flow cell. Automated cluster generation and paired-end sequencing with dual index reads was performed in a single 39-h run in a 2-bp × 251-bp.

### Phylogeny

The DNA polymerase gene was used for phylogenetic analysis. Amino-acid sequences were aligned using the MUSCLE software (Edgar, 2004). A phylogenetic tree was built using the MEGA 6.0 tool (Tamura et al., 2013) and the maximum likelihood method. In addition, we performed a hierarchical-clustering based on the gene presence/absence pattern of 5,443 NCVOGs, using the MeV tool (Eisen et al., 1998) with Pearson correlation as distance metric. The phylogenetic tree was visualized using the FigTree v1.4.1 tool^1^.

### Detection of the ORFan_850 Transcript by RT-PCR

Six hours after infection in *A. polyphaga* and in order to detect the transcript of putative ORFan (the ORF_850), RNA was extracted using RNeasy Mini kit (Qiagen, Hilden, Germany) after the pellet had been centrifuged at 9,000 × *g* for 15 min. DNA contamination was then checked by applying PCR using the HotStar TaqDNA polymerase kit (Qiagen). DNA contamination was removed from the RNA preparation using a TURBO DNA-Free kit (Ambion, Austin, TX, USA). The extracted volume was treated using RNeasy Mini Elute clean up Kit (Qiagen) and SuperScript VILO cDNA Synthesis Kit (Invitrogen, Carlsbad, CA, USA). The following primers were used to amplify the cDNA template by PCR with the Hot Star Taq DNA Polymarase (Qiagen): SV1 R 5′-TCTGAAACGTTATGTTCCGCA-3′, SV1 F 5′-TGCATTTTCCTTGGCACAAA-3′, SV2 R 5′-TCCGGATATCTTGGGCCATT-3′, SV2 F 5′-AGATGATGAATGTGTTCCTCCA-3′.

## Results

### Biological Features

**FIGURE 1:**
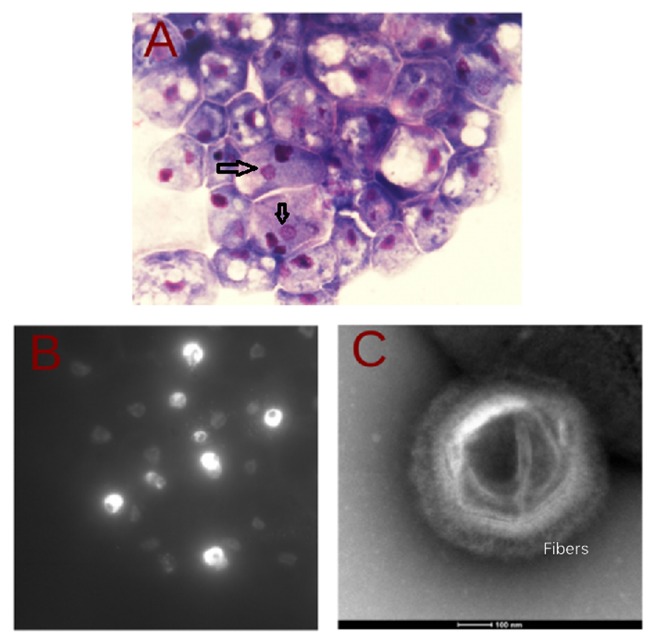
**Morphologic analysis of Saudi moumouvirus in *Acanthamoeba polyphaga.* (A)** Inverted microscopic observation of viral infection by hemacolor staining: the nucleus appears as dark purple spots; the virus factory is indicated by arrows (non-fluorescence image with 63×/1,4 oil lens, an exposure time of 64 ms). **(B)** DAPI: viral factories are seen as distinct, strongly stained patches (fluorescence image with a 63×/1,4 oil lens, an exposure time of 64 ms). **(C)** Negative staining electron microscopy of Saudi moumouvirus.

In this study, we isolated a new virus, known as SDMV by inoculating an *A. polyphaga* co-culture, from a sewage water sample taken before treatment (by filtration) at the King Abdulaziz University Hospital. It could also be grown in *A. griffini*, although not with *Acanthamoeba* sp. E4*, V. vermiformis* and *D. discoideum.* Presumptive morphologic observations showed a virus with a size of 500 nm, typical of the family of APMV (**Figure 1C**). The viral factory of SDMV was observed in the infected *A. polyphaga* cytoplasm with hemacolor and DAPI staining, respectively (**Figures 1A,B**). Some morphology features and virus factories appeared within the cytoplasm of *A. polyphaga* during the life cycle of the SDMV, similar to the other *Mimiviridae* members, and the morphology of SDMV factories (**Figures 1A,B**) were also similar to those previously observed for APMV*, Megavirus chiliensis* and *A. polyphaga moumouvirus* (Suzan-Monti et al., 2007; Arslan et al., 2011; Yoosuf et al., 2012). The size of the icosahedral capsid of SDMV with around 500 nm surrounded by a dense fiber layer, is comparable with members of family *Mimiviridae* (**Figure 1C**), such as APMV, moumouvirus and *Megavirus chiliensis* (500, 520, and 500 nm, respectively) (Klose et al., 2010; La Scola et al., 2010; Arslan et al., 2011; Yoosuf et al., 2012). In addition, the SDMV particles showed identifying, starfish-like vertex that resemble the other *Mimiviridae* members (Klose et al., 2010; Arslan et al., 2011).

Real-time PCR indicated that the SDMV belongs to mimiviruses lineage B.

### Genome Sequencing and Analysis

The SDMV genome assembly yielded two large contigs, with 174.294 and 855.762 bp KY110734). Its genome showed a low C+G% content (25.83%), which was similar to other mimiviruses (La Scola et al., 2003; Raoult et al., 2007; Assis et al., 2013). The SDMV genome encoded two tRNA molecules, tRNA-Cys (gca) and tRNA-His (gtg), as predicted in genomes of other mimiviruses from lineage B (Colson et al., 2013).

**FIGURE 2:**
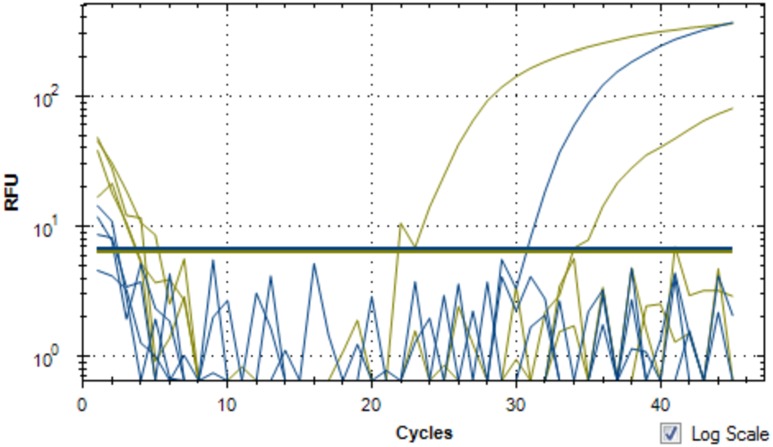
**qPCR of the SDMV ORFan_850 transcript in *Acanthamoeba castellanii* infected cells, 6 h post-infection**. The upper green and blue lines indicate the expression of ORFan 850 transcript with SV1 and SV2 designed primers pairs. SV1 primer pair reaction is indicated by the upper green line, SV2 primer pair reaction is indicated by the blue line, and the lower green line is the positive control of SDMV-DNA.

**FIGURE 3:**
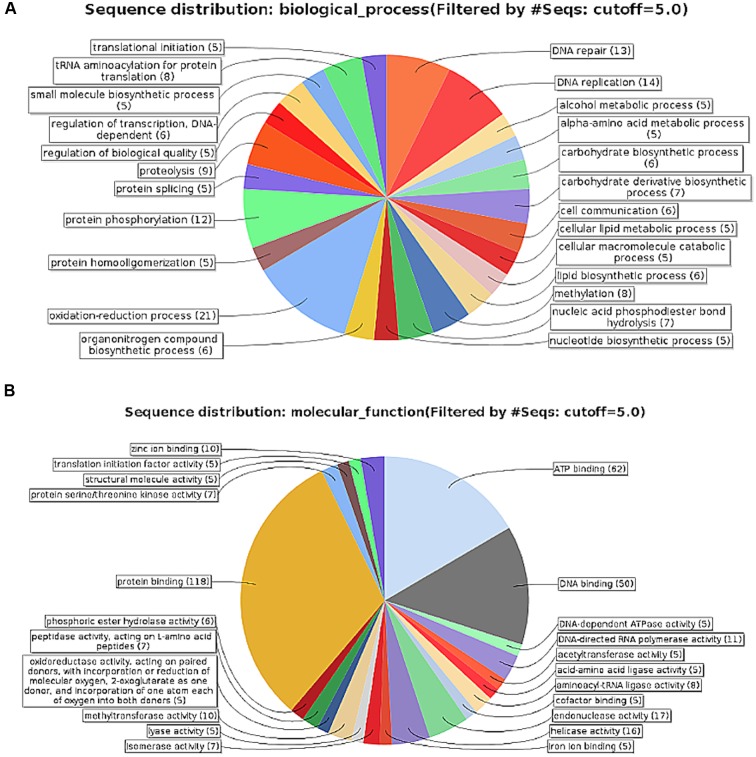
**Functional annotation of Saudi moumouvirus genome**. Characterization performed using the java-based free software Blast2go (available at http://www.blast2go.com/b2ghome). **(A)** Biological processes and **(B)** molecular functions assigned to the predicted SDMV genes, showing a large proportion of genes involved in nucleic acid processing and cellular metabolism, as well as viral morphogenesis and intracellular regulation. The cuttof of five was chosen to highlight the most representative biological processes and molecular functions groups.

The SDMV genome was predicted to encode a total of 953 ORFs, ranging in size from 34 to 2,914 amino acids (aa), with a mean size of 324 aa. Of them, 868 ORFs were functionally characterized by similarity analyses with previously reported sequences, ranging in size from 54 to 2914 aa (mean size: 349). Moreover, 40 ORFs were predicted without similarity with previously reported sequences, characterizing it as a putative ORFan. The transcription of ORFan L850 was confirmed by qPCR (**Figure 2**). In addition, 42 sequences had less than 100 aa without any predicted function (Pseudo-ORFs). The functional annotation of the SDMV genome revealed a great number of putative proteins related to several metabolic processes, nucleic acid binding and manipulation, as well as signal transduction processes (**Figure 3**). Curiously, we observed an unusual set of aminoacyl-tRNA synthetase (aaRS), which are translation-related genes not found in viruses other than mimiviruses. This new genome was predicted to encode nine aaRS molecules, including Methionyl, Isoleucyl, Asparaginyl (two sequences), Arginyl (two sequences), Cysteinyl (two sequences), and Tyrosyl-tRNA synthetase. In addition, around 40% (341/868) of sequences predicted in the SDMV genome were hypothetical proteins. Finally, the SDMV genome was predicted to encode 155 paralogous proteins (Coverage > 70%, identity > 30%, e-value < 1e-6), divided into 41 families, of which we highlight an ankyrin-like protein and an f-box/fnip (repeat containing) protein families, comprised of 32 and 20 sequences, respectively.

For the ORFs predicted in the SDMV genome, the best hits were most frequently proteins from mimivirus lineage B (97.7%). Moreover, a comparative analysis of the SDMV gene content against all mimivirus lineages revealed a higher mean identity with and bit-score distributions within mimivirus lineage B (**Supplementary Figure S1**).

### Detection of the ORFan_850 Transcript by RT-PCR

The amplified cDNA template, using real-time PCR with the Hot Star Taq DNA Polymarase, was positive (**Figure 2**), with the specific primers that indicated the presence of the ORFan gene (ORF_850), which was exclusive to the SDMV genome and has not been found in any other known organism, including other mimiviruses. The search and detection of such ORFan was performed as proof of concept of the presence of previously unreported sequences.

### Phylogeny

**FIGURE 4:**
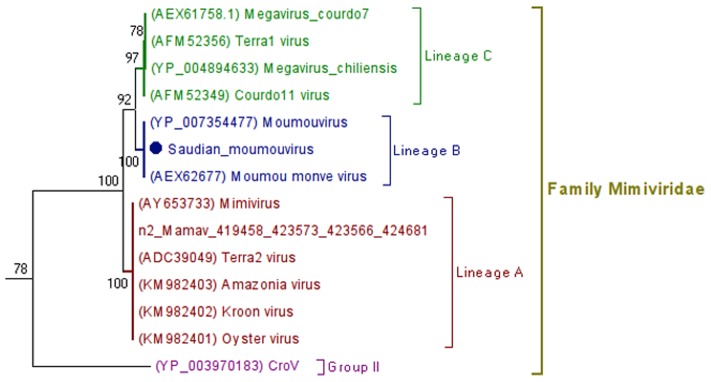
**β-DNA polymerase-based unrooted phylogenetic tree**. The analysis was performed using the MEGA 6.0 tool (maximum likelihood method and 1,000 bootstrap replicates) with predicted amino acid from the SDMV and other nucleocytoplasmic large DNA viruses (NCLDVs). The SDMV clusters with members of Mimivirus lineage B, which consists of Moumouvirus-like viruses. The values near the branches are bootstrap values and are used as confidence values for the tree branches.

**FIGURE 5:**
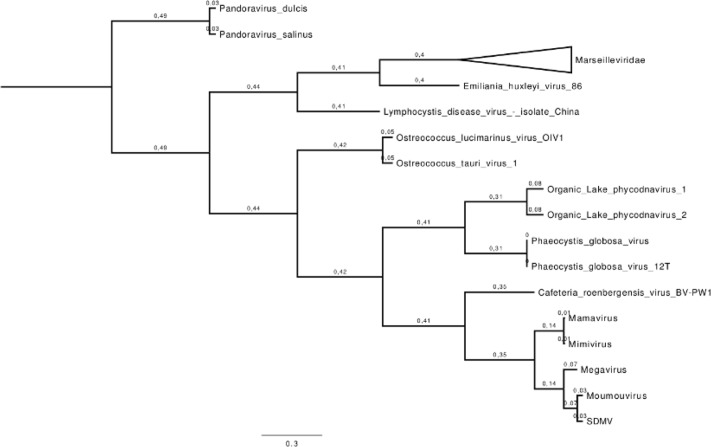
**Hierarchical clustering unrooted tree based on phyletic patterns**. Phylogeny based on the presence-absence matrix of 5443 NCVOG (clusters of orthologous genes shared by nucleo-cytoplasmic large DNA viruses). The phylogenetic tree was generated using the MeV 4.8.1 tool. The values near the branches are bootstrap values and are used as confidence values for the tree branches. Scale bar represent relative evolution distance.

β-DNA polymerase-based phylogenetic analysis corroborated all previous observations, clustering the SDMV isolate with members of family *Mimiviridae* lineage B, which includes *A. polyphaga* moumouvirus, Moumouvirus Monve and Moumouvirus Goulette strains (**Figure 4**). In addition, a hierarchical clustering tree (**Figure 5**), based on phyletic patterns, was constructed using a presence-absence matrix of 5,443 NCVOG (clusters of orthologous genes shared by NCLDVs). This phylogenetic tree was generated using the MeV 4.8.1 tool (Howe et al., 2010) and showed SDMV to be in the same branch of Moumouvirus (lineage B). These analyses corroborate all previous results suggesting the clustering of SMDV into mimivirus lineage B.

## Discussion

In this study, we report the isolation and characterization of SDMV from sewage water (pre-treatment), the first group B mimivirus isolated from Asia, after the recent first isolation of a group A in India (Chatterjee et al., 2016). In particular, there was no lysis indication of amoebal co-culture in the camel stool samples, although SDMV was revealed in the sewage water. This virus, infecting *A. polyphaga*, is closely related to members of the *Mimiviridae* group B, which consists of Moumouvirus-like viruses isolated in Europe and Africa. The SDMV strain has a capsid of 500 nm which can be observed by electron microscopy. This is at least 80 nm larger than other members of mimivirus lineage B (Pagnier et al., 2013). The SDMV showed a typical mimivirus morphology and viral factories. Previous studies have demonstrated that some specific virophages, such as Sputnik 3 and Zamilon virophages, can infect moumouvirus viral factories (Gaia et al., 2014). However, no virophages were detected in SDMV and amoebal co-culture and this new viral class remains undetected in Asia. Like all mimiviruses, SDMV was revealed to grow exclusively with *A. polyphaga* and *A. griffini*, but no lysis was observed with *A.* sp. E4*, V. vermiformis*, and *D. discoideum.*

Real-time PCR used for detection of mimiviruses of lineages A/B/C, marseilleviruses and virophages revealed that our sample SDMV belongs to mimiviruses lineage B.

The SDMV genome consists of a DNA molecule with around 1,030,056 bp, which is larger than all previously described moumouvirus-like genomes (Yoosuf et al., 2012; Pagnier et al., 2013). Even when partially sequenced, this genome was predicted to encode to 953 ORFs (considering putative ORFs, ORFans and Pseudo-ORFs), more than the gene content of Moumouvirus type species (Yoosuf et al., 2012). Colson et al. (2013) reported the presence of three tRNA molecules encoded by moumouvirus-like viruses for histidine, cysteine and leucine, while SDMV was predicted to encode for only two such molecules (histidine and cysteine). In addition, no differences were observed when we compared the codon and amino acid usage of SDMV and other mimiviruses (data not shown).

Furthermore, functional annotation revealed a distinct set of aaRS when compared to moumouviruses. It was not possible to identify a Methionyl-tRNA synthetase gene in SDMV, an important translation-related molecule encoded by all known mimiviruses. Moreover, the SDMV encoded only two copies of Arginyl-tRNA synthetase, while Moumouvirus encoded four (Colson et al., 2013). Curiously, the presence of duplicated Arginyl-tRNA synthetase genes was not related to increased amino acid usage. In addition, no duplication in the Cysteinyl-tRNA synthetase gene has previously been reported in mimiviruses lineage B, but the biological effect of such duplication remains unclear given that cysteine is poorly used by all known mimiviruses. The SDMV was predicted to encode two molecules of Asparaginyl-tRNA synthetase, which was previously predicted only in Megavirus chilensis (Group C). Finally, we detected 48 sequences with no similarity to any sequence available in the NCBI nr database. The qPCR performed for one of these sequences (ORF_850) highlighted the potential of other ORFans to be transcriptionally active, supporting the need for additional work to describe their unpredicted functions.

The mean amino acid identity analysis suggest a close relationship among SDMV and group B mimiviruses (average amino acid identity: 97%), although BLAST analyses has evidenced substantial divergences suggesting the establishment of SDMV as a new mimivirus group B isolate. The SDMV showed 845 blast hits with moumouvirus (NC_020104.1), but only 49% of identical sequences. The remaining hits presented more than 10,000 mismatches distributed among proteins from several functional groups such as structural and regulatory proteins, host range proteins, metabolic-related protein, and others. Such divergences can be the result of its particular evolutionary history in such environment. The divergences among the SDMV and mimiviruses from lineages A and C are even higher, being observed an average amino acid identity revolving around 60%, with few identical sequences. These observations highlight a probable long evolutionary history since the splitting of mimivirus lineages. The phylogenetic analyses were performed using two distinct approaches: (1) β-DNA polymerase-based phylogenetic analysis, which used a canonical molecular target subject to different methods (NJ and ML), and (2) phyletic-based phylogenetic analysis, which used a presence-absence matrix of genes encoded by all analyzed viruses, using the hierarchical clustering method to delineate the evolution of this group based on gene gain and loss profile. In both strategies, the SDMV were grouped into mimivirus Group B with other moumouvirus-like viruses, which corroborates all previous data regarding the characterization of SDMV. Until recently, the detection of moumouvirus-like viruses was restricted to Europe, South America and Africa, with a low detection rate when compared to other mimivirus groups, mainly mimivirus Group A. In terms of the diversity of mimivirus circulation in Asia, we believe that SDMV is only the tip of iceberg, and new areas and environments must be investigated to uncover the actual diversity and spread of such viruses, which could contribute to understanding their ecology and evolution.

## Author Contributions

BLS designed the experiments; EA sent the samples; LB, FA, and JA analyzed the data; LB, PJ, and CR performed the experiments; LB, FA and JA wrote the manuscript; DR reviewed and corrected the manuscript.

## Conflict of Interest Statement

The authors declare that the research was conducted in the absence of any commercial or financial relationships that could be construed as a potential conflict of interest.

## Footnotes

1 http://tree.bio.ed.ac.uk/software/figtree/

## Supplementary Material

FIGURE S1 **Comparative analysis of Saudi moumouvirus virus gene content with other mimivirus sequences**. The analysis showed the highest identity and bit-score distributions of Saudi moumouvirus against mimivirus group B sequences, such as moumouvirus **(B)**. Moreover, the similarity decreased toward *Acanthamoeba polyphaga mimivirus* (APMV) **(A)** and Megavirus chilensis **(C)** of groups A and C, respectively. At the top of figures are mean and median of each compared group, considering identity and bit score distribution.

## Abbreviations

SDMV: Saudi moumouvirus
