# Supplementary figures and images for: Saudi Moumouvirus, the First Group B Mimivirus Isolated from Asia
Source: Front Microbiol. 2016 Dec 20;7:2029. doi: 10.3389/fmicb.2016.02029 (PMC5167723; doi:10.3389/fmicb.2016.02029)

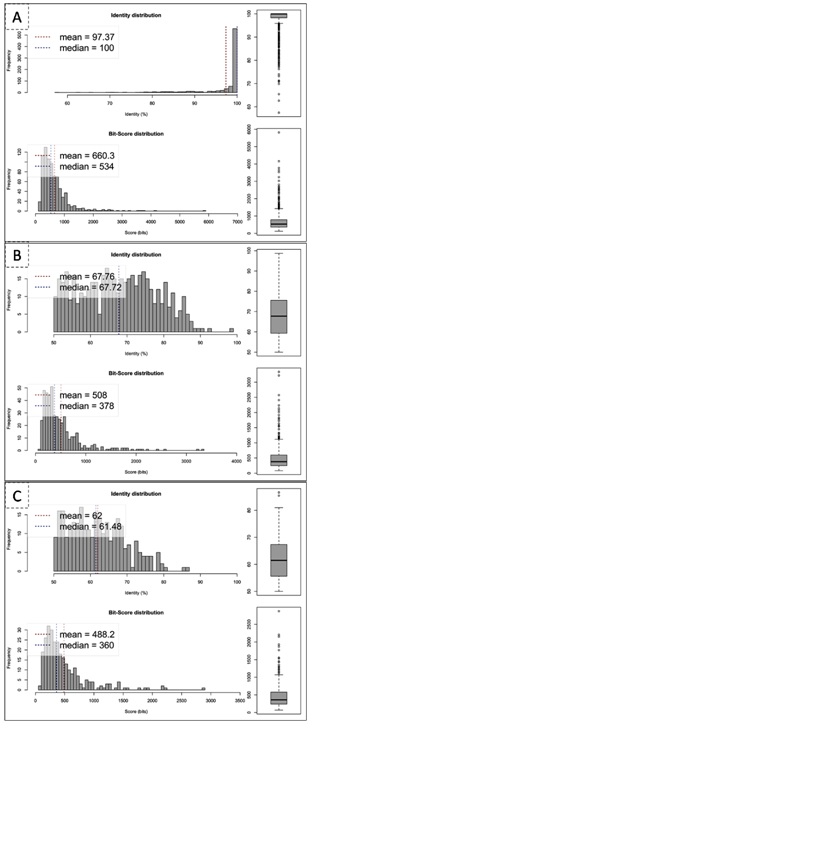

Supplement: FIGURE S1 — Comparative analysis of Saudi moumouvirus virus gene content with other mimivirus sequences. The analysis showed the highest identity and bit-score distributions of Saudi moumouvirus against mimivirus group B sequences, such as moumouvirus (B). Moreover, the similarity decreased toward Acanthamoeba polyphaga mimivirus (APMV) (A) and Megavirus chilensis (C) of groups A and C, respectively. At the top of figures are mean and median of each compared group, considering identity and bit score distribution. [file Image_1.JPEG]
